# Polish version of the Aesthetic Experience Questionnaire: validation and psychometric characteristics

**DOI:** 10.3389/fpsyg.2023.1214928

**Published:** 2023-08-31

**Authors:** Agata Hiacynta Świątek, Małgorzata Szcześniak, Karolina Wojtkowiak, Michał Stempień, Marianna Chmiel

**Affiliations:** Faculty of Social Sciences, Institute of Psychology, University of Szczecin, Szczecin, Poland

**Keywords:** aesthetic experience, measurement, engagement with art, aesthetic competence, artistic creative activities

## Abstract

**Introduction:**

The purpose of the article is to present the results of works on the Polish version of the Aesthetic Experience Questionnaire (AEQ). The AEQ is a 22-item tool for assessing aesthetic experience in the following dimensions: emotional, cultural, perceptual, understanding, and two dimensions about flow (proximal conditions and flow experience).

**Methods:**

In the course of works on the Polish version of the AEQ, 3 independent studies with the participation of more than 800 people were carried out. In addition to the AEQ measurement, the tools included: the Emotion Regulation Strategies for Artistic Creative Activities Scale, the Brief Music in Mood Regulation, the Aesthetic Competence Scale, the Aesthetic Processing Preference Scale, the Need for Cognition Scale, the Center for Epidemiologic Studies - Depression Scale, the Material Values Scale and the Multidimensional Existential Meaning Scale.

**Results:**

The results obtained in the three studies through Confirmatory Factor Analysis indicated the compliance of the factor structure of the Polish version of the AEQ with the original and its good psychometric characteristics. It was also shown that the overall result and individual components of the aesthetic experience correlate positively with emotion regulation through artistic creative activities and mood regulation through music, aesthetic competences (music, literature, plastic arts, film), cognitive curiosity and some dimensions of aesthetic processing preferences. The studies also proved a very weak positive relationship between aesthetic experience and meaning of life. The assumption about a negative correlation between aesthetic experience and depression or materialism was not confirmed.

**Discussion:**

The Polish version of the AEQ is a credible psychometric measurement and encourages scientists to design research on the psychology of art and aesthetics in the Polish cultural conditions.

## Introduction

In Poland, the psychology of art and aesthetics is not a popular research topic, which is reflected in the lack of psychometric tools to conduct research in this area. Therefore, the main issue discussed in the article is the validation of a new tool – the Aesthetic Experience Questionnaire (AEQ; Wanzer et al., 2020) for the Polish cultural conditions. The work presents the issues and the current knowledge of aesthetic experience (AE), and describes the process of developing and verifying the psychometric characteristics of the Polish version of the AEQ. It draws attention to the AEQ’s structure, analyzing it with the use of the Confirmatory Factor Analysis (CFA) and answering in this way to the authors’ suggestions which indicated the need for such a verification in future research. The CFA was carried out separately on data from three studies in order to confirm whether the results were coincidental and whether the tool structure is actually confirmed in the Polish reality. Then, the assumptions about the relationships between aesthetic experience and its potential correlations were verified. The results of these three separate studies, their limitations and indications concerning further work, are summarized in the discussion.

According to Marković (2012), aesthetic experience is one of the least clarified concepts in the psychology of art and experimental aesthetics. Similarly, Brattico et al. (2013) note that despite the presence in the literature of research on experiencing aesthetic stimuli, the “aesthetic experience” concept is relatively poorly defined in psychology. These words remain relevant after more than 10 years. Meng and Liang (2022) write that the subjective and objective attribute of AE remains unclear, and they search for stable, reliable grounds for the nature of AE in aesthetic anthropology and neuroaesthetics. Marković (2012, p. 1) writes: “Generally, aesthetic experience can be defined as a special state of mind that is qualitatively different from the everyday experience.” He gives readers insight into three components of AE: aesthetic fascination, aesthetic appraisal, and aesthetic emotion. He notices that AE does not occur automatically, but it results from the individual’s attitude to the external environment and social context. Everyday objects or works of art will not elicit AE in all people. As Vessel et al. (2012) write, aesthetic experience is connected with the integration of reactions resulting from sensory perception, emotional reactions and personal meaning. On the one hand, it is recognized that reacting to aesthetic stimuli is rooted in human biology. There is a well-known study in which a team of three Italian researchers used the fMRI technique to analyze the biological reactions of viewers who were not experts in response to masterpieces of classical and renaissance sculpture (Di Dio et al., 2007). From a medical point of view, scientists have been able to explain the physiological grounds for aesthetic experience based on neurological studies for more than 20 years (Ramachandran and Hirstein, 1999; Vessel et al., 2012). On the other hand, apart from biological mechanisms, full aesthetic experience seems to require more complex forms of thinking. Bullot and Reber (2013) in their article proposed a psycho-historical framework for the science of art appreciation and tried to bridge the gap between researchers of the perception of art from different fields of science.

While developing their method of measuring aesthetic experience (AEQ), Wanzer et al. (2020) used theoretical frameworks proposed by Csikszentmihalyi and Robinson (1990) based on interviews with employees of museums. Referring to the results of their research, the authors indicate four dimensions of AE connected with works of art. These are the perceptual, emotional, cognitive, and communicative dimensions. The first of them concerns the composition of the work and the aesthetic aspects of its composition (color, texture, form of the painting or sculpture). The second dimension covers different (pleasant and unpleasant) emotions which occur in response to contact with the work of art. The third dimension is the understanding of the work of art through the impact of knowledge of its cultural significance and historical knowledge (general knowledge of art, the artist, and the specific work). The fourth dimension concerns the meaning of the artist’s intentions in creating the aesthetic experience, i.e., communication of the work function (interpretation of the artist’s message by a viewer). The authors also proposed two dimensions connected with the flow experience – the occurrence of proximal conditions as well as the flow experience itself as a result of coming into contact and engagement in contemplation of the work of art.

The Aesthetic Experience Questionnaire (AEQ; Wanzer et al., 2020) is a psychological measurement which evaluates the intensity of individual components of aesthetic experience during the contemplation of a work of art. Although it was constructed mainly in relation to visual arts (such as paintings and sculptures), the authors of the original version do not exclude its application to other fields of art, for example music. In such a case, they suggest adjusting the selected items to the specific nature of the given field of art. 22 statements were grouped into 6 dimensions: emotional (e.g., My emotions change as I continue to view the work of art), cultural (e.g., I compare the past culture of the art with present-day culture), perceptive (e.g., I focus on the subtle aspects of the work of art), understanding (e.g., I gain new insights about the work of art itself), proximal flow conditions (e.g., I have a clear idea of what to look for when viewing works of art) and the flow experience during contact with works of art (e.g., I lose track of time when I view works of art). The study subjects assess how much they agree (or disagree) with each of the statements, using a 7-point Likert scale (from 1 = “I definitely disagree” to 7 = “I definitely agree”). In the original study (Wanzer et al., 2020), all AEQ dimensions were associated with each other and the overall AEQ score, ranging between the values *r* = 0.240 and *r* = 0.719. Most of the correlations were at the moderate level (less than *r* = 0.500). The results for each of the six dimensions are obtained by summing the points gained by the study subject in each scale: emotional (items 1–4), cultural (items 5–8), perceptive (items 9–11), understanding (items 12–15), proximal flow conditions (items 16–18) and the flow experience during contact with works of art (items 19–22). Moreover, the overall result is calculated by adding up the points gained by the study subject in subscales. The questionnaire does not contain reversed questions or buffer questions.

Works on the Polish validation of the AEQ scale constituted part of a larger study conducted based on the consent of the Committee for the Ethics of Research of the Institute of Psychology at the University of Szczecin (KB 6/2022 of 27/04/2022). In the first stage of developing the Polish version, three independent translations from English into Polish were commissioned. Based on these translations, a Polish version was prepared jointly by the researchers. This translation was consulted with an art expert. Next, the scale was subjected to reverse translation. After verifying the consistency of the reverse translation with the original, 25 people were initially asked to complete it. These people assessed the clearness and correctness of the individual statements. They also had the possibility to submit their own comments. As no reservations were raised, this Polish version was placed in the questionnaire batteries. In order to exclude the impact of the order of completing the tools by the respondents on the assessment of the tool’s psychometric properties, in individual studies, the AEQ was placed at the beginning, in the middle and at the end of the questionnaire batteries.

### Assumptions adopted

While developing their method of measuring aesthetic experience (AEQ), Wanzer et al. (2020) analyzed the relationships between the results obtained by their study subjects in the AEQ and the level of the intensity of openness to experience, inspiration, curiosity and exploration. There is no other measure of the same construct in Poland, nor anything similar enough to check convergent validity. Moreover, if we wanted to replicate the variables from the original study, it would be difficult, because we do not have their Polish versions. Therefore, in our study, we explore the topic, proposing correlates which were not considered by Wanzer and her team. Our studies were aimed at bringing new knowledge of aesthetic experience in addition to the knowledge that already exists. That is why the emphasis in the first study was put on measurements connected with art and the function of aesthetic experience in human life. The following paragraphs present hypotheses and their justification in order to explain the purposefulness of the selection of particular variables.

Using music to regulate the mood was taken into consideration as the first variable. According to Granot et al. (2021), there are many purposes of listening to music, e.g., experiencing aesthetic pleasure, determining one’s own identity, bringing back memories or changing one’s own mood. Osowiecka and Gacka (2016) write that while creating music presenting hardships (e.g., hip hop brings up the topics of addictions and poverty), performers cope in this way with problems and give listeners hope for a better future. Furthermore, they refer to the work of Van den Tol and Edwards (2015), who believe that the identification with emotions from a song allows listeners to re-experience something, the experience of connecting with the message of the song may motivate them to be active (undertake actions), the aesthetic value may divert their attention from problems, and memories evoked by the piece of music allow them to experience them again and see them in a new light. Of course, the processing of visual and musical stimuli differs from each other; the very specificity of these stimuli is different (the sculpture is static, the musical work is changing in time). However, referring to the concept of the aesthetic quotient (Dan et al., 2021), which consists of competences in separate fields of art, it can be assumed that if someone is “aesthetically intelligent” in one field of art, he or she is probably better able to engage in the reception of other artistic domains and use them in an adaptive way (emotion regulation). That is why it was assumed that people who have the ability to become emotionally and cognitively involved to a great extent in aesthetic experience are very likely to listen to music in order to change their mood:

*H1*: The aesthetic experience and its individual dimensions positively correlate with using music in order to regulate mood.

The next variable was the regulation of emotions through artistic creative activities. Also in this case, it was assumed that people who have strong aesthetic experience are more willing to apply strategies of coping with difficulties through their own artistic creativity. Fancourt et al. (2020) showed in their studies that training in doing an artistic activity, regular engagement in the production of art, and enjoyment while engaging are all associated with a greater ability to use artistic activities to regulate our emotions. Osowiecka and Gacka (2016, p. 69) write that “most artists are convinced (and certainly they have such a feeling) that they create to experience emotional well-being” and they draw attention to the human need to create art even in extremely unfavorable conditions, e.g., by concentration camp prisoners or political prisoners. Based on the above intuitions, it was assumed that:

*H2*: The aesthetic experience and its components positively correlate with the regulation of emotions through artistic creative activities and their individual types.

The sense of the meaning of life was the next variable that we wanted to analyze in the AE context. Art frequently touches on universal, timeless motives and themes connected with the physical or mental reality experienced by the author. The viewer interprets the message of the work of art, referring not only to cultural norms but also to personal systems of meanings or specific situations from their own life. Contact with an aesthetic object may stimulate reflection going beyond the aesthetic situation (e.g., in the case where viewing works of art created several centuries ago raises the question of what we and our society leave behind when we pass away). It was exploratorily assumed that a high level of engagement in aesthetic experience (particularly in the aspect of understanding and culture) may help to understand and discover the purpose and meaning of one’s own existence. Therefore, it was assumed that:

*H3*: Aesthetic experience, together with its dimensions, positively correlates with the sense of meaning of life.

The frequency of contact with artistic objects or participation in artistic events may sensitize people to artistic experiences. For people who rarely participate in cultural events, the situational context and circumstances may play an important role (the sense of uniqueness of the whole situation, attitude, and conviction of seeing “something unusual” may prevail over conscious focus on the reception of the work). People who are familiar with the arts often visit places intended for art exhibitions and are likely to focus more easily on the art object itself. They “immerse” in the work which makes their experience in contact with a specific artwork more reflective, intense, richer, than in people who, for example, rarely visit a museum, go there because of prestige or as tourists.

Moreover, the intensity and depth of perceiving artworks probably also varies depending on the role that art plays in a person’s life. People whose paid work is related to the arts (whether they are artists or sellers of art) naturally need to update their art-related competencies and are more sensitive to various aspects of art. By concentrating on the work, they have a chance for stronger experiences, at least in terms of perceiving and understanding art. Similarly, a higher level of aesthetic experience may be characteristic of people who give art an important place in their lives in the category of passion/hobby. It is a way of spending time and personal development dictated by one’s choice, curiosity, the desire to aesthetically experience the world or the feeling of discovering something. Based on these insights, we suppose that this is why the frequency of participation in artistic events and the meaning given to art may differentiate art recipients in terms of the intensity of aesthetic experiences related to art.

*H4*: Groups with different levels of participation in artistic activities (frequency groups) and giving different meanings to artistic activities (meaning groups) differ in aeasthetic experience.

In the second study, we asked about the relationships between AE and aesthetic competences, aesthetic processing preferences, and the need for cognition.

Most likely, people who declare stronger aesthetic experience may also be more competent recipients of a given field of art or several fields of art. The exposure to art and aesthetic experience based on it are conducive to building aesthetic competences. At the same time, a high level of aesthetic quotient could make it easier initially to get engaged in aesthetic experience. It is known, for example, that experts are more differentiated and flexible in the assessment of works of art than people who have little knowledge of art. Moreover, it was stated that experts in general gave higher assessments in almost all scales connected with the appreciation of art (Leder et al., 2012). The studies of Fayn et al. (2018) indicate that deeper engagement of people with wider knowledge of art in the reception of art is connected with more detailed aesthetic experiences. Hence, according to the fifth hypothesis:

*H5*: Aesthetic experience and its dimensions positively correlate with aesthetic competences in four fields of art.

Kopatich et al. (2021) write that motivation to become engaged in controlled art processing increases interest in and knowledge of art. Based on reports of other researchers, the same authors indicate that people more willing to deal with aesthetic objects better appreciate complexity and feel greater pleasure while viewing works of art. In their own studies, two dimensions of the aesthetic processing, i.e., propensity to contextualize and appreciation of complexity, co-occurred with interest in art and knowledge of art, while intolerance for ambiguity negatively correlated with knowledge of art. On this basis, it was assumed that:

*H6*: Aesthetic experience and its dimensions positively correlate with appreciation of complexity in art and propensity to contextualize artworks, while they negatively correlate with intolerance for ambiguity in art.

The need for cognition leads to “controlled intellect,” and people with a high level of this feature are prone to carefully consider available information (Madrid and Patterson, 2016). The inclination for analytical observation of reality may encourage greater engagement in contemplation of art. It may be assumed that in the case of people with a high level of the need for cognition, contact with art will be connected with stronger aesthetic experience, therefore:

*H7*: Aesthetic experience and its dimensions positively correlate with the need for cognition.

While planning the third study, independent of the others, we wondered whether there are negative correlations for aesthetic experience. What features or conditions may be unfavorable for intensive engagement in the reception of art? For the analysis, we selected materialistic values and depression indicators.

According to the results obtained by Diessner et al. (2008), the higher the person’s engagement in beauty, the less probable it is that they are materialistic. On this basis, it may be assumed that a materialistic attitude to life and finding happiness in possession are not conducive to engagement in the reception of art. If works of art are not treated as investments or material security, the “food for thought” (aesthetic experience) will not be “nourishing” for people who hold materialistic values. Therefore, it is assumed that:

*H8*: Aesthetic experience and its dimensions negatively correlate with following materialistic values in life.

Depression is an illness that can lead to immense suffering and results in the reduction of activity in daily life (Blomdahl et al., 2013). Diessner et al. (2008) write that depression is negatively correlated with the overall factor of becoming engaged in beauty and with two subscales. There are also data indicating that engagement in art and culture may be a protective factor, as it is connected with a reduced frequency of the occurrence of depression in the population (Elsden and Roe, 2021). Therefore, it is assumed that:

*H9*: Aesthetic experience and its dimensions negatively correlate with depression indicators.

After presenting the above-described hypotheses, we will present three independent studies in which we analyze the structure of the Polish version of the AEQ and the nomological network of its correlations.

## Study 1

### Participants

The participants were 402 Polish adults (71% women). The age range of the sample was 18–72 years (*M* = 25.33; *SD* = 9.69). They were asked about the frequency of artistic activity on a 5-point Likert scale, where 1 = less or not at all, 2 = several times a month, 3 = 1–2 times a week, 4 = several times a week, and 5 = daily or almost daily. Most of them (32%) declared daily or almost daily artistic activity, followed by several times a month (22%), less or not at all (17%), several times a week (16%), and 1–2 times a week (13%). A question was also added about what artistic activity means to the respondent. More than half of the people answered that artistic activity is a passion for them, which they do in their free time from work or study (57%). For the second group of participants, it is something they are learning or developing with a view to earning money in this area (25%). The third group represents people for whom artistic activity is not important (13%). Only 5% of the respondents admitted that they make a living from artistic activity. All the participants were recruited through non-probability convenience sampling and expressed informed and written consent to participate in Studies 1–3.

### Procedure, data and statistical analysis

Before performing a CFA on the data, the skewness and kurtosis values of all 22 AEQ items were considered, to ensure the assumption of a normal distribution required by the structural equation. Although there is no explicit cut-off to denote a symmetric distribution (Bowen and Guo, 2011), the accepted ±2 cut-off was considered as not a cause for concern (Field, 2000).

In order to examine the adequacy of the hypothesized model, a variety fit indices (Swanson and Holton, 2005; Flynn et al. 2019) were measured: insignificant χ^2^ test (*p* > 0.05), the ratio χ^2^/df (≤ 5), goodness-of-fit index (GFI), Tucker-Lewis index (TLI), and the comparative fit index (CFI) ≥ 0.90, standardized mean square residual (SRMS) ≤ 0.06, root mean square error of approximation (RMSEA), LO, and HI ≤ 0.08 (Hooper et al., 2008). The internal consistency of the six factors and overall AEQ was assessed, adopting the criterion of at least α > 0.75 as a general standard for an acceptable value (Weiner and Greene, 2017). Moreover, we used the corrected item–total correlation to check the consistency between responses to an item and the sum of the other items (Furr and Bacharach, 2008). For loading estimates, the following rule of thumb was assumed: 0.71–excellent, 0.63–very good, 0.55–good, 0.45–fair, and 0.32–poor (Harrington, 2009).

The construct validity of the AEQ and its potential correlates was assessed in Study 1 through the use of the Brief Music in Mood Regulation Scale, the Emotion Regulation Strategies for Artistic Creative Activities Scale, and the Multidimensional Existential Meaning Scale. The basis for the selection of these variables was the assumption that they would correlate positively with aesthetic experience, as some of the constructs are conceptually related or inversely associated. In order to be clear when describing and interpreting the correlation coefficients and their strength, we adopted the following framework: weak between ±0.1 and ±0.3, moderate between ±0.4 and ±0.6, and strong between ±0.7 and ±0.9 (Akoglu, 2018).

To determine the suitable sample size, an *a priori* power analysis was performed, using G*Power 3.1.9.4 with a bivariate normal model correlation (Faul et al., 2007). A small effect size of 0.20, an alpha of 0.05, and a power of 0.95 were assumed. The result of the analysis showed that a total sample size would require around 266 participants. The justification for using the value of Pearson’s *r* = 0.20 is based on general recommendations that, in individual differences research, such a benchmark is justified (Brydges, 2019).

A one-way analysis of variance (ANOVA) was carried out to check whether belonging to groups with different levels of participation in artistic activities (frequency groups) and giving different meanings to artistic activities (meaning groups) differentiates aeasthetic experience (H4). A Levene’s test was used to verify the assumptions of data homoscedasticity with a significance level of *p* < 0.05. A Tukey’s *post hoc* test or a Games-Howell *post hoc* test were then computed, depending on the homogeneity of variance value. The Tukey test output was examined when the assumption of homogeneity of variance was not violated (Levene’s test not significant). Instead, the Games-Howell test was considered when this assumption was violated (Leven’s test significant; Allen, 2017).

The research project (Studies 1–3) was conducted according to the recommendations of the Declaration of Helsinki. Statistical analyses were performed using SPSS Statistics for Windows, version 20, and IBM SPSS AMOS 21.

### Measures

The Brief Music in Mood Regulation Scale (B-MMR; Saarikallio, 2012) assesses the degree to which the study subject uses music to regulate their own emotions and estimates the degree to which the study subject uses individual strategies. It consists of 21 statements grouped into 7 strategies of modifying the mood, which in the current study proved to be very reliable, both for the individual subscales and for the overall result: entertainment (α = 0.89), revival (α = 0.88), strong feelings (α = 0.89), distraction (α = 0.86), relieving oneself (α = 0.86), intellectual work (α = 0.88), solace (α = 0.92), and overall mood regulation (α = 0.95). The study subjects take a position on each statement using a 5-point scale, where the “always/almost always” answer is equivalent to the study subject receiving 5 points, while one point is assigned for the “almost never/never” answer. To obtain the results for each individual mood regulation strategy, it is necessary to sum the points received by the study subject from the statements included in that factor. The overall result is obtained by summing all points (α = 0.95). The Polish translation of the tool was used in the study.

The Emotion Regulation Strategies for Artistic Creative Activities Scale (ERS-ACA; Fancourt et al., 2019) is an 18-item tool to measure engagement in artistic activities as a measure regulating emotions. It provides a calculation of the result for overall engagement in artistic activities as a strategy of dealing with emotions and a reflection of the intensity of using the following distinguished strategies: avoidance strategies (α = 0.92), approach strategies (α = 0.90), self-development strategies (α = 0.90), and overall emotion regulation (α = 0.95). The scale does not concern any specific art domain – the study subject is asked to think about their favorite artistic activity. Then the respondent reads each statement and refers to it, ticking the answer on a 5-point Likert scale (1 = “I definitely disagree,” 5 = “I definitely agree”). The Polish translation of the scale was used in the study.

The Multidimensional Existential Meaning Scale (MEMS) is a self-report instrument developed by George and Park (2017). The Polish adaptation was created by Gerymski and Krok (2020). The scale measures meaning in life in three different dimensions: comprehension (e.g., I know what my life is about), purpose (e.g., I have aims in my life that are worth striving for), and mattering (e.g., I am certain that my life is of importance). The respondents assess each of the 9 statements by using answers on a 7-point Likert scale that ranges from 1 – “very strongly disagree” to 7 – “very strongly agree.” The higher the overall score obtained, the higher the level of the comprehension, purpose and mattering in life. The reliability in the individual subscales was sufficient: comprehension (α = 0.75), purpose (α = 0.65), mattering (α = 0.83), as well as in overall multidimensional existential meaning (α = 0.91).

### Results

Table 1 presents the means, standard deviations, values of skewness and kurtosis, CFA loadings, and corrected item-total correlations for the aesthetic experience items. The skewness and kurtosis values did not exceed ±2.

**Table 1:** Descriptive statistics for AEQ items, CFA loadings, and corrected item-total correlations (*N* = 402).

Item	*M*	*SD*	Skewness	Kurtosis	Loadings	Corrected item-total correlations
AEQ1	4.60	1.99	−0.39	−1.01	0.71	0.74
AEQ2	4.62	1.86	−0.41	−0.86	0.93	0.78
AEQ3	4.54	1.94	−0.38	−0.97	0.89	0.78
AEQ4	3.76	1.96	0.19	−1.15	0.87	0.61
AEQ5	4.06	2.08	−0.07	−1.32	0.85	0.64
AEQ6	3.94	2.02	−0.07	−1.30	0.79	0.72
AEQ7	3.64	2.11	0.15	−1.37	0.85	0.61
AEQ8	4.20	2.02	−0.24	−1.20	0.77	0.77
AEQ9	4.51	1.97	−0.37	−1.10	0.89	0.76
AEQ10	4.81	2.01	−0.55	−0.97	0.89	0.75
AEQ11	4.59	1.97	−0.49	−0.97	0.86	0.81
AEQ12	4.40	1.93	−0.26	−1.04	0.83	0.79
AEQ13	4.50	1.94	−0.36	−1.02	0.91	0.79
AEQ14	4.48	1.94	−0.42	−0.96	0.86	0.86
AEQ15	4.47	2.04	−0.37	−1.13	0.84	0.78
AEQ16	3.77	1.89	0.13	−1.11	0.87	0.67
AEQ17	3.93	1.83	−0.02	−1.01	0.88	0.71
AEQ18	4.10	1.88	−0.08	−1.07	0.77	0.76
AEQ19	3.52	2.02	0.27	−1.17	0.85	0.70
AEQ20	3.89	1.00	0.02	−1.21	0.92	0.78
AEQ21	3.97	1.99	−0.08	−1.19	0.92	0.80
AEQ22	4.65	1.97	−0.46	0.98	0.83	0.83

AEQ, aesthetic experience questionnaire.

The AEQ subjected to the CFA confirmed the factor structure obtained by Wanzer et al. (2020) through Exploratory Factor Analysis in their original article about the development of the AEQ. All standardized loadings in the CFA model were above 0.70 (between 0.71 and 0.93), indicating excellent values for the AEQ items. The test for goodness-of-fit showed that the specified model had a six-factor structure consisting of emotional (items 1–4), cultural (items 5–8), perceptual (items 9–11), understanding (items 12–15), flow conditions (items 16–18), and flow experience (items 19–22), and represented an acceptable fit with the data: χ^2^ = 804.91, df = 194, *p* = 0.000, χ^2^/df = 4.149, GFI = 0.84, TLI = 0.91, CFI = 0.92, SRMS = 0.04, RMSEA = 0.08, LO = 0.08, HI = 0.09. Although χ^2^ was significant, this statistic is sensitive to sample size, which for Study 1 was 402. Also, the GFI was slightly below 0.90. However, these statistics tend to present a downward bias with a large number of degrees of freedom (Hooper et al., 2008). Considering all the other results, the model was accepted in its present form. The internal reliability presented via Cronbach’s alpha and composite reliability (CR) for the six factors was as follows: emotional (α = 0.90; CR = 0.91), cultural (α = 0.89; CR = 0.89), perceptual (α = 0.91; CR = 0.91), understanding (α = 0.92; CR = 0.92), flow conditions (α = 0.87; CR = 0.88), flow experience (α = 0.93; CR = 0.93), and overall aesthetic experience (α = 0.96; CR = 0.98). With respect to the correlations among the six dimensions of the AEQ, they ranged from 0.61 to 0.79 (*p* < 0.001), presenting moderate and strong associations. The strongest link was that between the perceptual dimension of the AEQ and understanding.

We obtained positive and varied correlations in terms of strength (Table 2) between the aesthetic experience overall / its six dimensions and mood regulation overall / its seven dimensions. They were mostly significant weak, except for a few that were closed to moderate, and two insignificant values (between: AEQ cultural and B-MMR discharge; AEQ perceptual and B-MMR discharge). Similar results were obtained between aesthetic experience overall / its six dimensions and multidimensional existential meaning / its three dimensions. Moderate associations were observed between aesthetic experience overall / its six dimensions and emotion regulation strategies for artistic creative activities / its three dimensions.

**Table 2:** Correlations between dimensions/overall score of AEQ, B-MMR, ERS-ACA, and MEMS (*N* = 402).

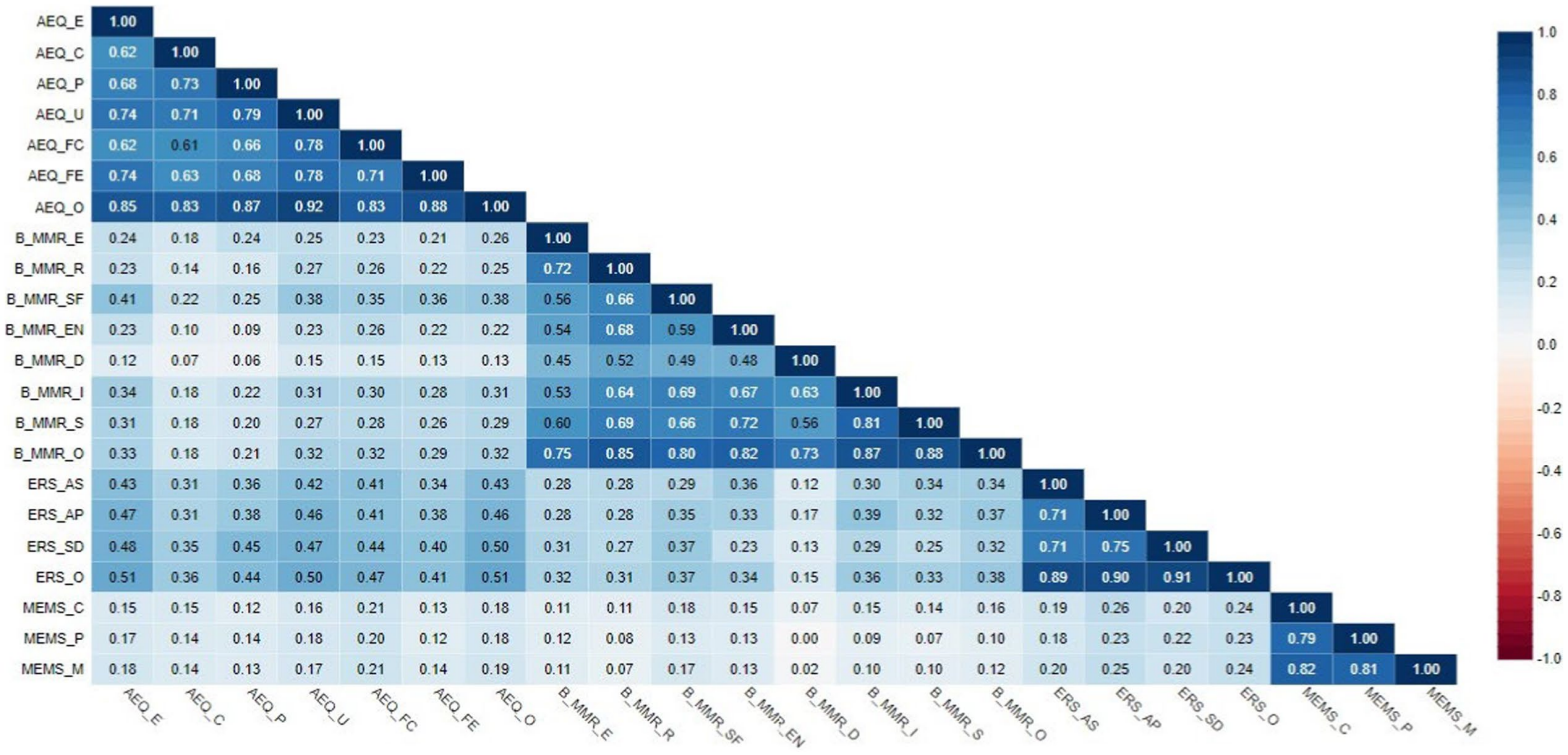

AEQ-E, aesthetic experience emotional; AEQ-C, cultural; AEQ-P, perceptual; AEQ-U, understanding; AEQ-FC, flow proximal conditions; AEQ-FE, flow experience; AEQ-O, overall; B-MMR-E, escapism; B-MMR-R, revival; B-MMR-SF, strong feelings; B-MMR-EN, mood regulation entertainment; B-MMR-D, discharge; B-MMR-I, introspection; B-MMR-S, solace; B-MMR-O, overall; ERS-AS, emotion regulation avoidance strategies; ERS-AP, approach; ERS-SD, self-development; ERS-O, overall; MEMS-C, existential meaning comprehension; MEMS-P, purpose; MEMS-M, mattering; MEMS-O, overall.

The results on one-way ANOVA suggest that there were statistically significant differences for scores on the AEQ between frequency groups [*F*_(401,4)_ = 5.441, *p* < 0.001] and between meaning groups [*F*_(401,3)_ = 9.447, *p* < 0.001]. Since the Levene statistic for frequency groups (2.295, *p* = 0.059) showed no violation of the assumption of equality means (*p* > 0.05), the Tukey’s outcomes were considered. The mean differences in aesthetic experience were noticed between participants who declared participating less or not at all in artistic activities and two other frequency groups: participants practicing artistic activities several times a month (*MD* = −20.40; *p* < 0.001) and participants practicing aesthetic activities daily or almost daily (*MD* = −21.24; *p* < 0.001). More precisely, people for whom artistic activities have little or no importance presented levels of aesthetic experience significantly lower (*M* = 77.72) than those who practice artistic activities several times a month (*M* = 98.12) and daily or almost daily (*M* = 98.96). There were not other significant differences in aesthetic experience between abovementioned frequency groups and those people who practice artistic activities 1–2 times a week (*M* = 88.86) and several times a week (*M* = 93.96). In turn, the Levene statistic for meaning groups (2.778, *p* = 0.041) showed violation of the assumption of equality means (*p* < 0.05). Hence, the Games-Howell’s outcomes were examined. The mean differences in AE were observed between respondents who acknowledged that artistic activity was not important for them and two other meaning groups: participants learning or developing with a view to earning money in art (*MD* = −28.12; *p* < 0.001) and participants who considered artistic activity as a passion for them (*MD* = −19.60; *p* < 0.001). Thus, it can be assumed that people who are not interested in art (*M* = 71.05) tend to score significantly lower in aesthetic experience than people who work within field of art (*M* = 99.17) and who are passionate about artistic activity (*M* = 95.38). These meaning groups did not differ in aesthetic experience from the respondents who admitted that they make a living from artistic activity (*M* = 90.66).

## Study 2

### Participants

The participants were 201 Polish adults (65% women). The age range of the sample was 18–76 years (*M* = 26.40; *SD* = 11.89). The respondents were asked about their level of education. Most of them were still studying (48%), followed by those who had completed higher education (33%), secondary education (9%), diploma of vocational technician (6%), primary education (3%), and basic vocational education (1%).

### Measures

The Aesthetic Competence Scale (ACS) is a tool created by a research team led by Dan et al. (2021). In the current study, we used its Polish translation. The instrument measures aesthetic quotient and consists of 20 items, which are divided into four subscales: music (e.g., While listening to music, I can make a certain judgment about the music, performers, and composers), visual art (e.g., I can identify the style and genre of a painting), literature (e.g., I may indulge in the plot of the story while reading a book), and film (e.g., I enjoy the beauty brought forth by films). The participants respond to all statements using a 5-point Likert scale (from 1 – “strongly disagree” to 5 – “strongly agree”). The higher the overall score obtained, the higher the aesthetic quotient. The internal reliability for the four factors were as follows: music (α = 0.90), visual art (α = 0.89), literature (α = 0.91), film (α = 0.92), and overall aesthetic competence (α = 0.96).

The Aesthetic Processing Preference Scale (APPS; Kopatich et al., 2021) is a scale that measures individual differences in the scope of the controlled processing in relation to aesthetics. It consists of 13 statements belonging to 3 factors. They are: appreciation of complexity, intolerance for ambiguity, and propensity to contextualize. The set of answers for the study subject consists of 6 possible answers, from “I definitely disagree” to “I definitely agree.” One point is assigned to the answer “I definitely disagree,” while 6 points are assigned to the answer “I definitely agree.” The scale does not contain reversed questions. The results are added up and interpreted separately within three subscales, by adding up the points gained by the study subject within each factor. The internal reliability for the three factors and overall result was as follows: appreciation of complexity (α = 0.91), intolerance for ambiguity (α = 0.79), and propensity to contextualize (α = 0.88), overall aesthetic processing preference (α = 0.89).

The Need for Cognition Scale (NCS) is a self-report tool used to measure the tendency to engage in and enjoy effortful cognitive tasks. The scale created by Cacioppo and Petty (1982) was adapted into Polish by Matusz et al. (2011). The one-dimensional scale consists of 36 items (e.g., I find it especially satisfying to complete an important task that required a lot of thinking and mental effort). The participants assess each statement by using answers on a 5-point Likert scale (from 1 – “strongly disagree” to 5 – “strongly agree”). The higher the overall score obtained, the higher the level of need for cognition. The internal reliability for the whole scale was α = 0.86.

### Results

Table 3 shows selected descriptive statistics for the aesthetic experience items. All standardized loadings in the CFA model were very good (between 0.61 and 0.91). Like in Study 1, the test for goodness-of-fit in Study 2 confirmed a six-factorial structure of the AEQ: χ^2^ = 447.79, df = 194, *p* = 0.000, χ^2^/df = 2.308, GFI = 0.82, TLI = 0.92, CFI = 0.93, SRMS = 0.04, RMSEA = 0.08, LO = 0.07, and HI = 0.09.

**Table 3:** Descriptive statistics for AEQ items, CFA loadings, and corrected item-total correlations (*N* = 201).

Item	*M*	*SD*	Skewness	Kurtosis	Loadings	Corrected item-total correlations
AEQ1	4.35	1.96	−0.16	−1.18	0.89	0.79
AEQ2	4.32	1.91	−0.34	−0.97	0.90	0.79
AEQ3	4.20	1.89	−0.13	−1.13	0.89	0.77
AEQ4	3.20	1.82	0.55	−0.68	0.73	0.62
AEQ5	3.76	2.01	0.11	−1.24	0.72	0.57
AEQ6	3.92	1.98	0.08	−1.25	0.77	0.68
AEQ7	3.88	2.04	0.06	−1.31	0.81	0.65
AEQ8	4.19	1.89	−0.12	−1.02	0.82	0.69
AEQ9	4.35	1.88	−0.24	−0.94	0.86	0.79
AEQ10	4.85	1.89	−0.64	−0.74	0.79	0.73
AEQ11	4.36	1.93	−0.42	−0.95	0.89	0.84
AEQ12	4.45	1.90	−0.34	−1.02	0.85	0.79
AEQ13	4.76	1.89	−0.55	−0.77	0.85	0.75
AEQ14	4.17	1.84	−0.26	−0.93	0.86	0.80
AEQ15	4.13	1.95	−0.13	−1.13	0.61	0.62
AEQ16	3.55	1.80	0.24	−0.93	0.70	0.65
AEQ17	3.99	1.76	−0.01	−0.93	0.88	0.73
AEQ18	4.00	1.80	−0.03	−0.99	0.91	0.78
AEQ19	3.55	2.02	0.26	−1.13	0.81	0.68
AEQ20	3.84	2.05	0.12	−1.22	0.86	0.74
AEQ21	3.97	1.93	0.03	−1.15	0.87	0.78
AEQ22	4.46	1.93	−0.25	−0.11	0.89	0.83

AEQ, aesthetic experience questionnaire.

The internal reliability for the six factors was as follows: emotional (α = 0.91; CR = 0.91), cultural (α = 0.86; CR = 0.86), perceptual (α = 0.88; CR = 0.89), understanding (α = 0.86; CR = 0.87), flow conditions (α = 0.88; CR = 0.87), flow experience (α = 0.91; CR = 0.92), and overall aesthetic experience (α = 0.96; CR = 0.98). The correlations among the six dimensions of the AEQ, which ranged from 0.59 to 0.77 (*p* < 0.001), showed moderate and strong associations. Like in Study 1, the strongest link was between the perceptual dimension of the AEQ and understanding.

Aesthetic experience showed significant positive and moderate correlations with visual art, overall aesthetic competence, appreciation of complexity, propensity to contextualize, and overall aesthetic processing preference. Moreover, significant positive associations were observed between aesthetic experience and aesthetic competence in music, literature, and film, intolerance for ambiguity, and need for cognition (Table 4).

**Table 4:** Correlations between dimensions/overall score of AEQ, ACS, APPS, and NCS (*N* = 201).

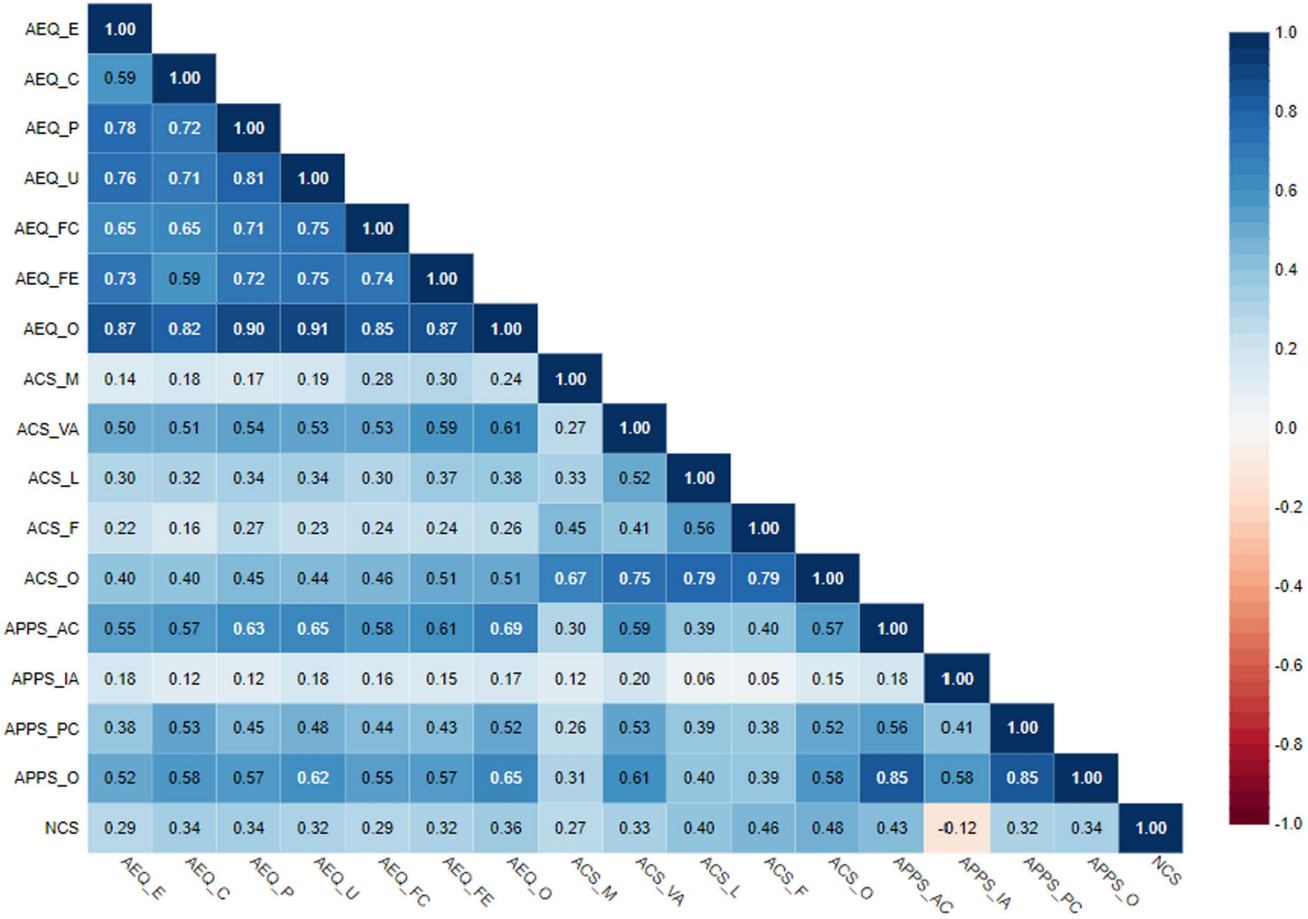

AEQ-E, aesthetic experience emotional; AEQ-C, cultural; AEQ-P, perceptual; AEQ-U, understanding; AEQ-FC, flow conditions; AEQ-FE, flow experience; AEQ-O, overall; ACS-M, aesthetic competence music; ACS-VA, visual art; ACS-L, literature; ACS-F, Film; ACS-O, overall; APPS-AC, aesthetic processing appreciation of complexity; APPS-IA, intolerance for ambiguity; APPS-PC, propensity to contextualize; APPS-O, overall; NCS, need for cognition.

## Study 3

The aim of the third study was to test divergent validity of the AEQ. Since divergent validity is the demonstration of a weak correlation of a new validated measure with tools that measure other variables (Heale and Twycross, 2015), depression and materialism were selected as variables negatively correlated with engagement with beauty, a construct somewhat similar to aesthetic experiences. Both tools are quite well established in research in Polish cultural conditions.

### Participants

The sample consisted of a total of 210 Polish adults (71% women). The age range of all the participants was 18–74 years (*M* = 26.87; *SD* = 11.97). Similarly to Study 2, the respondents were asked about their level of education. A relatively large proportion of the participants were still studying (52%), followed by those who had completed higher education (29%), secondary education (8%), diploma of vocational technician (6%), primary education (3%), and basic vocational education (2%).

### Measures

The Center for Epidemiologic Studies Depression Scale (CES-D) is a self-report tool used to measure depressive symptoms. The scale was created by Radloff (1977) and adapted by Jankowski (2016). The instrument consists of 20 items, which are divided into four components of depressive symptomatology: somatic symptoms (e.g., I was bothered by things that usually do not bother me), depressed affect (e.g., I felt that I could not shake off the blues even with help from my family or friends), positive affect (e.g., I felt I was just as good as other people), and interpersonal relations (e.g., I felt that people dislike me). The participants answer how often over the past week they experienced symptoms associated with depression using a 3-point Likert scale from 1 – “some or little of the time” to 3 – “most or almost all the time.” Scores range from 0 (no depressive symptoms) to 60 (maximum depressiveness). The higher the overall score obtained, the greater the depressive symptoms. The reliability of the whole scale was α = 0.84 and for its four subscales was: somatic symptoms (α = 0.83), depressed affect (α = 0.89), positive affect (α = 0.81), and interpersonal relations (α = 0.65).

The Material Values Scale (MVS) is a self-report tool developed by Richins and Dawson (1992). The Polish adaptation was created by Górnik-Durose (2016). The instrument measures valuing the possession and accumulation of material goods by three factors: success (e.g., I admire people who own expensive homes, cars, and clothes), centrality (e.g., Buying things gives me a lot of pleasure), and happiness (e.g., My life would be better if I owned certain things I do not have). The respondents relate to all statements using a 5-point Likert scale (from 1 – “strongly disagree” to 5 – “strongly agree”). The higher the overall score obtained, the greater the appreciation of material values. The reliability of the whole scale was α = 0.82 and for its three subscales was: success (α = 0.65), centrality (α = 0.57), and happiness (α = 0.67).

### Results

Table 5 presents the descriptive statistics for the aesthetic experience items. All standardized loadings in the CFA model were very good (between 0.67 and 0.93). The test for the goodness-of-fit test in Study 3 indicated a poor fit to the data: χ^2^ = 542.00, df = 194, *p* = 0.000, χ^2^/df = 2.794, GFI = 0.81, TLI = 0.90, CFI = 0.91, SRMS = 0.05, RMSEA = 0.09, LO = 0.08, and HI = 0.10.

**Table 5:** Descriptive statistics for AEQ items, CFA loadings, and corrected item-total correlations (*N* = 210).

Item	*M*	*SD*	Skewness	Kurtosis	Loadings	Corrected item-total correlations
AEQ1	4.55	1.89	−0.37	−0.88	0.90	0.80
AEQ2	4.57	1.79	−0.44	−0.79	0.89	0.78
AEQ3	4.46	1.86	−0.29	−1.00	0.93	0.81
AEQ4	3.33	1.80	0.37	−0.93	0.67	0.58
AEQ5	4.01	1.93	0.09	−1.20	0.75	0.59
AEQ6	4.13	2.03	−0.11	−1.35	0.80	0.59
AEQ7	3.70	1.87	0.13	−1.15	0.70	0.52
AEQ8	4.16	1.83	−0.18	−1.00	0.77	0.75
AEQ9	4.51	1.91	−0.38	−0.98	0.88	0.77
AEQ10	4.97	1.89	−0.80	−0.46	0.88	0.74
AEQ11	4.49	1.95	−0.31	−1.08	0.87	0.80
AEQ12	4.51	1.88	−0.29	−0.99	0.86	0.82
AEQ13	4.63	1.88	−0.53	−0.77	0.83	0.74
AEQ14	4.41	1.96	−0.28	−1.11	0.90	0.84
AEQ15	4.38	1.90	−0.32	−1.07	0.74	0.70
AEQ16	3.60	1.78	0.21	−0.95	0.73	0.66
AEQ17	3.77	1.71	0.13	−0.80	0.86	0.69
AEQ18	3.91	1.78	−0.01	−1.02	0.90	0.74
AEQ19	3.57	2.02	0.28	−1.18	0.71	0.69
AEQ20	3.94	1.95	−0.03	−1.19	0.82	0.74
AEQ21	4.13	1.81	−0.12	−0.96	0.89	0.82
AEQ22	4.68	1.97	−0.48	−0.92	0.92	0.84

AEQ, aesthetic experience questionnaire.

The internal reliability for the six factors was as follows: emotional (α = 0.91; CR = 0.91), cultural (α = 0.84; CR = 0.84), perceptual (α = 0.91; CR = 0.91), understanding (α = 0.90; CR = 0.90), flow conditions (α = 0.86; CR = 0.91), flow experience (α = 0.92; CR = 0.88), and overall aesthetic experience (α = 0.96; CR = 0.98). The correlations among the six dimensions of the AEQ, which ranged from 0.59 to 0.76 (*p* < 0.001), presented moderate and strong associations. The strongest link was between flow experience and understanding.

Some dimensions of aesthetic experience showed a few weak negative correlations with happiness related to the possession and accumulation of material goods (Table 6). The other variables displayed no significant associations with aesthetic experience.

**Table 6:** Correlations between dimensions/overall score of AEQ, CES, and MVS (*N* = 210).

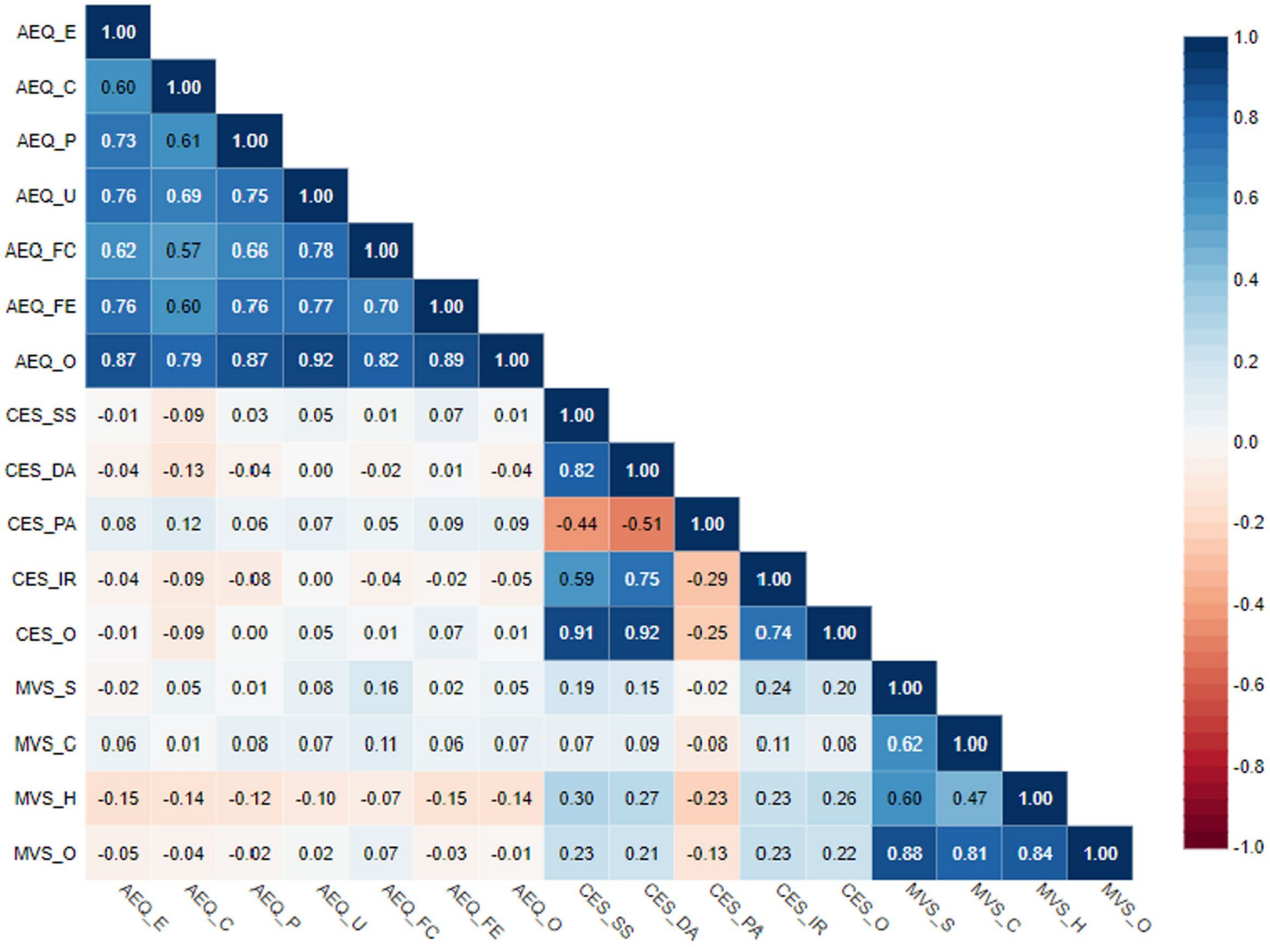

AEQ-E, aesthetic experience emotional; AEQ-C, cultural; AEQ-P, perceptual; AEQ-U, understanding; AEQ-FC, flow proximal conditions; AEQ-FE, flow experience; AEQ-O, overall; CES-SS, center for epidemiologic studies depression somatic symptoms; CES-DA, depressed affect; CES-PA, positive affect; CES-IR, interpersonal relations; CES-O, overall; MVS-S, material values success; MVS-C, centrality; MVS-H, happiness; MVS-O, overall.

## Discussion

The article describes (to the best of our knowledge) the first attempt to validate the Aesthetic Experience Questionnaire in a foreign language. After finding articles citing the publication of Wanzer et al. (2020), none of them concerned the validation, adaptation or creation of a version in a foreign language. In order to adjust the AEQ to the Polish conditions, 3 studies were conducted with 9 main hypotheses. It is interesting that, in the original study, the authors did not perform a CFA. Therefore, in order to confirm the non-randomness of the factor division for the AEQ, in this study, a CFA was carried out three times, in each of the three independent attempts.

With respect to the structure of the AEQ, the results obtained in the three studies support the six-factor model of the original AEQ, obtained through the Exploratory Factor Analyses. The good fit indices of the CFA suggest the psychometric solidity of the Polish version of the AEQ. The reliability values, like for the original ones, confirm the internal consistency of the measure. Thus, the results denote that the Polish version of the scale is a reliable tool and manifests similar psychometric characteristics to Wanzer’s version. Moreover, the correlations between all six subscales presented comparable results, although being slightly stronger. Therefore, the AEQ, with its emotional, cultural, perceptual, understanding, flow conditions, and flow experience dimensions, can be used to assess aesthetic experience.

The literature indicates various types of tool validity verification, including content validity, construct validity, face validity and criterion validity (Bahariniya et al., 2021). The latter includes predictive, concurrent, convergent and discriminant validity. Due to the difficulty of checking the convergent validity of the AEQ (Polish researchers do not have many fully verified measurements from the scope of the psychology of aesthetics and art), we selected only variables potentially positively and negatively correlated with it. When selecting potential correlations, it was decided to regulate emotions using art [specific for music (H1), and generally for artistic creative activities in any field (H2)], as well as aesthetic competences in four selected fields of art (H5). The relationship between the cognitive aspects of the perception of art (controlled processing) and the intensity of aesthetic experience was considered (H6), and the need for cognition was taken into account (H7). With knowledge of the therapeutic significance of art, the hypothesis on a positive relationship between aesthetic experience and the meaning of life was formulated (H3). The concept of beauty plays an essential role in aesthetics; therefore, referring to knowledge of engagement in beauty (Diessner et al., 2008), it was assumed that aesthetic experience will be negatively correlated with materialism (H8) and depression (H9) to check discriminant validity.

In the first study, it was proven that the overall result and individual components of aesthetic experience positively correlate with mood regulation through music (H1). It can be said that people who have strong, deep aesthetic experience are in general more prone to use music to modify their emotions. The weakest correlations for all dimensions of the AEQ are two strategies of using music, i.e., discharge (venting negative emotions) and distraction from worries and stress. In the case of discharge and distraction, no relationship with the perceptual dimension of the AEQ was observed. Also, the strategy of using music based on discharge does not correlate with the cultural dimension of the AEQ. The results obtained are not particularly surprising. People who use music to distract themselves from difficulties or to give vent to anger do not need the ability to focus on details of the work or to reflect on the cultural context of the work for this purpose. A further issue is that music (especially popular music) has become westernized (Drygas, 2015) and the ability to analyze the work of art in the cultural context is not necessary to use music to cope with difficult emotions.

Moreover, it was confirmed that aesthetic experience is positively correlated with emotion regulation through artistic creative activities (H2). The relationships between individual dimensions of both tools range from moderate to weak, while the dimension of practicing creative activities for self-development is most closely correlated with all dimensions of the AEQ. This means that people who declare stronger aesthetic experience in contact with art are more likely to undertake creative activities to cope with everyday challenges. The relationships observed are statistically significant, both in the case of strategies based on self-development, obtaining new insight into the situation, and in the case of avoiding difficulties (distracting attention from them). A question arises whether it would be possible to distinguish profiles of people applying the given strategies more frequently and the manner in which they are connected with aesthetic experience, to which currently we do not have an answer. We are of the opinion that the results fit into the generally available knowledge of the therapeutic role of art. Shaping artistic sensitivity and building resources of aesthetic experience may be conducive to coping with difficulties in a creative and constructive manner. Even in the case of the dominance of avoidance strategies, performing creative activities seems to be a more psycho-physically hygienic strategy of coping with difficulties than running away from problems into, for example, a virtual world or drugs.

Furthermore, the assumption about the co-occurrence of strong results in the AEQ and a stronger overall result and in subscales for meaning of life (H3) was also confirmed. Nevertheless, the correlation observed is very weak. We are willing to assume that although engagement in art may be a source of pleasant emotions and may give the recipient of the work grounds for reflections going beyond the aesthetic situation, the sense of understanding, purpose and meaning of one’s own life depend to a greater extent on other psychological factors. Art may be a tool that supports searching for meaning, but most likely, if it is not in the center of the individual’s interests (profession, passion), the role of aesthetic experience for the sense of the meaning of life is marginal.

With respect to H4, the results proved to be consistent with the adopted assumptions. First, it can be said that the frequency of participation in artistic events tends to be associated with stronger and deeper aesthetic experiences. In fact, the frequency of participation turned out to differentiate some frequency groups in relation to the AEQ. Although our justification for the hypothesis seems logical in the light of contact with art as a goal itself, we are not sure why this is the case. The frequency of participation in cultural events may be driven by many motives – from purely cognitive to social (being invited or spending time with friends) or these related to prestige. The type of artistic event can also be important for the depth and type of experience – at a concert of popular music, the aesthetic experiences of the musician and the listener are related to the context of community, as well. The reception of the painting during the vernissage can reflect an immersion into the finished work. Second, it can be suggested that giving different meanings to artistic activities may be related to aesthetic experiences. In fact, the strongest aesthetic experiences are shared by people who learn or develop with a view to earning money in art and those who consider art as their passion. However, it is difficult to say unequivocally whether it is the more intense way of receiving art that predestines to deal with art professionally or out of passion, or rather it is the other way around – that making art the “axis” of life determines the depth of artistic experience.

In the second study, 3 hypotheses were verified. According to the assumptions adopted, all dimensions of aesthetic experience correlated significantly and positively with the overall result in the ACS (the level of the Aesthetic Quotient in relation to four fields of art) and the result for each of the dimensions separately (H5). The AEQ measurement was created in relation to visual arts, which is why it is not surprising that all AEQ factors were most closely connected with aesthetic competences in the scope of visual arts (the strength of these relationships is moderate), and then with the overall result of the ACS (also moderate). The results suggest that people with high aesthetic competences also declare stronger cognitive and emotional aesthetic experience. We assume the existence of a two-way relationship, which means that: acquiring greater competences in the scope of art makes people more sensitive to details of works, allows them to better understand those works and to experience more intensive contact with art, but also vice versa, being receptive to aesthetic experience will deepen the aesthetic competences.

Practical implications from the results obtained refer rather to artistic education at the general level – it is difficult to acquire knowledge of art without the possibility of having close contact with art. In Poland, the “cultural knowledge” subject (combining anthropological issues with history of art) was removed from compulsory subjects and teaching it at schools depends on the school management’s decision. Currently, a student of a secondary school or technical secondary school may choose from music, art and philosophy (taught at the first grade, 1 h per week, within the 4-year cycle of the secondary-school education). In the situation where artistic subjects are treated as a “necessary evil,” it is difficult to build aesthetic competences and knowledge of cultural heritage. Teachers do not have time to go with students to museums, art exhibitions, the theater or concerts. The aesthetic competences and sensitivity of young recipients are shaped mainly through mass culture. It is difficult to acquire aesthetic competences if a person does not experience art in real life, if the contact with art is very rare and reserved only for elites (and due to this, understood to a very limited extent) or marginalized (considered as an insignificant “addition” in the general education).

The next hypothesis subject to the verification (H6) concerned aesthetic experience with three elements of aesthetic processing preferences. All dimensions of the AEQ significantly positively correlate with the appreciation of complexity. Similarly, all dimensions of the AEQ significantly positively correlate with the propensity to contextualize. It may be assumed that people who declare that they have strong aesthetic experience also appreciate the high level of complexity of works of art, have the ability to look at the work of art from many perspectives and like ambiguity. It is also not surprising that in contact with works of art, they are willing to take into account a broader context (the person of the artist, the time during which the work was created and the circumstances). This is also indicated by the positive verification of hypothesis 7 (H7; concerns the relationship between AE and the need for cognition). For people who like to engage in deep reflective thoughts, contemplating complex works of art can provide a stronger aesthetic experience and be cognitively satisfying. The assumption about the negative correlation of the AE dimensions with intolerance for ambiguity was not confirmed. It was observed that there are very weak positive correlations among the emotional dimension, understanding, proximal conditions for flow, flow experience, and intolerance for ambiguity. The result also indicates that people who prefer explicitness in the interpretation and simpler aesthetic objects may also have stronger aesthetic experience. It may be said that less sophisticated aesthetic tastes do not make it impossible to derive deeper experience from contact with art, although in the case of the cultural and perceptual dimension of AE, the relationship with intolerance for ambiguity does not occur at all. In the case of preferring structurally and symbolically uncomplicated works of art, the ability to notice details and to refer the work to works of other artists or times of creation probably is not significant or necessary.

The third study verified two assumptions concerning potentially negative correlations of aesthetic experience, i.e., materialistic attitude (H8) and depression (H9). When it comes to the measurement of the depression indicator, no significant relationships between the CES-D overall result and the AEQ overall result and individual dimensions were observed. Also, the analysis of the relationships between the results obtained from the study subjects in the individual factors of both tools proved that there are no significant relationships.

Similarly, no significant relationship between the MVS overall result and the AEQ overall result and subscales was found. Some statistically significant correlations between the dimension of materialistic happiness and the emotional dimension, cultural dimension, flow experience and the AEQ overall result were observed, although they were very weak negative correlations. People whose sense of happiness is rooted in possession and material well-being declare to a lesser extent that they have aesthetic experience. Aesthetic experience is closer to the spiritual sphere; it does not translate into the increase in material resources and it is therefore probably not perceived as valuable by people with a materialistic value orientation. Hence, people appreciating material values probably do not attach much importance to it and do not get engaged in experiencing art. It does not mean that materialists do not appreciate art at all; it probably interests them if it can be treated as an investment or if possessing it is connected with prestige. Also, no relationships between the central character of material values and the dimensions of aesthetic experience were observed, while a very weak positive relationship between material success and proximal conditions for flow is surprising. High results on the scale of material success indicate that people appreciating material values base their sense of success on possessing things that are expensive and make an impression on other people. The criterion of conditions making it easier to experience flow is connected, e.g., with possessing a clear vision of details of the work of art to which attention should be paid and the conviction of the correctness of one’s own reflections on the work of art. For people who are interested in art for materialistic reasons (collection or investments), certain knowledge of the art market, current trends and fashion and the value of other works of a given artist are necessary when they purchase works of art. Therefore, such a person may have specific expectations toward works of art and, from this point of view, an apparently unexpected correlation seems to be justified.

## Limitations

In terms of the methodology, the researchers in some cases using Polish translations of certain tools from the scope of art and aesthetics instead of their validations may be considered a fault of this study. We would like to mention that studies were conducted which confirm the good psychometric characteristics of the Polish versions of the ACS, B-MMR and ERS-ACA, and articles concerning these measurements are currently being prepared by Świątek and her research team.

The description of the results lacks statistics comparing groups of professional artists, amateur artists, and people who do not deal with art. People with different levels of knowledge and practice in the scope of art participated in the studies, but uneven representation would make it difficult to obtain reliable results. In the future, this could be taken into account.

Carried out online, the three studies are burdened with all the reservations characteristic for this type of studies (from the randomness of the participant group to difficulty in providing the study subjects with identical conditions when completing the questionnaire).

Another point is that the selection of correlation variables did not reflect, in terms of criteria, the AEQ factors (for example, no tool to measure dispositional flow was applied). It seems to us that the novelty was also an advantage and constitutes an added value to the validation process. Some of the measured constructs seem to be similar to AE (e.g., the ACS measuring the level of the Aesthetic Quotient for music, visual arts, literature and film contained questions about knowledge, understanding of art and emotional engagement, which are distinguished as dimensions in the AEQ), but the strength of the correlations does not indicate that they overlap.

## Conclusion

The article probably describes the first validation of the Aesthetic Experience Questionnaire for the Polish cultural conditions. Moreover, it is probably the first publication in which a CFA was carried out for this tool. The results of 3 independent studies indicate that the Polish version of the AEQ has very good psychometric characteristics and is highly recommendable for research on the psychology of aesthetics and art as well as individual differences (the Polish version of the questionnaire is available in the Appendix). Additionally, the studies demonstrated the existence of statistically significant, positive correlations between AE and mood regulation through art (listening to music, artistic creative activities), aesthetic competences, need for cognition, some dimensions of aesthetic processing preference, and also to a much lesser extent existential meaning. Also, negative – although very weak – relationships between one of the three dimensions of materialism (happiness based on material values) and the AEQ overall result and three dimensions were observed.

## Data availability statement

The datasets presented in this study can be found in online repositories. The names of the repository/repositories and accession number(s) can be found at: https://osf.io/rsby2/?view_only=24860fa200e44fd09976b78f5b92eab0.

## Ethics statement

The studies involving humans were approved by the Committee for the Ethics of Research of the Institute of Psychology at the University of Szczecin (KB 6/2022 of 27/04/2022). The studies were conducted in accordance with the local legislation and institutional requirements. The participants provided their written informed consent to participate in this study.

## Author contributions

AŚ and MSz conceived and designed the study, analyzed the data, interpreted the results, drafted the manuscript, wrote the manuscript, read, and approved the final version. KW, MSt, and MC performed the study, analyzed the data, drafted the manuscript, read, and approved the final version. All authors contributed to the article and approved the submitted version.

## Conflict of interest

The authors declare that the research was conducted in the absence of any commercial or financial relationships that could be construed as a potential conflict of interest.

## Publisher’s note

All claims expressed in this article are solely those of the authors and do not necessarily represent those of their affiliated organizations, or those of the publisher, the editors and the reviewers. Any product that may be evaluated in this article, or claim that may be made by its manufacturer, is not guaranteed or endorsed by the publisher.
