# Supplementary material for: Polish version of the Aesthetic Experience Questionnaire: validation and psychometric characteristics
Source: Front Psychol. 2023 Aug 31;14:1214928. doi: 10.3389/fpsyg.2023.1214928 (PMC10501565; doi:10.3389/fpsyg.2023.1214928)
Supplement: Supplementary file 1 [file Data_Sheet_1.docx]

**Appendix**

**Polish Version of The Aesthetic Experience Questionnaire (AEQ)**

**Kwestionariusz Doświadczeń Estetycznych (KDE)**

(Świątek, Szcześniak, Wojtkowiak, Stempień, Chmiel, 2023)

In general, when I view art…

**Zazwyczaj, kiedy oglądam sztukę…**

|  | Zdecydo-wanie się nie zgadzam | Nie zgadzam się | Raczej się nie zgadzam | Ani się zgadzam, ani nie zgadzam się | Raczej się zgadzam | Zgadzam się | Zdecydo-wanie się zgadzam |
| --- | --- | --- | --- | --- | --- | --- | --- |
| 1. I experience a wide range of emotions.  **Doświadczam szerokiego zakresu emocji.** | 1 | 2 | 3 | 4 | 5 | 6 | 7 |
| 2. My emotions change as I continue to view the work of art.  **Moje emocje ulegają zmianie w trakcie oglądania dzieła sztuki.** | 1 | 2 | 3 | 4 | 5 | 6 | 7 |
| 3. I feel moved.  **Czuję się poruszony/a.** | 1 | 2 | 3 | 4 | 5 | 6 | 7 |
| 4. I experience a physical reaction.  **Doświadczam reakcji fizycznej.** | 1 | 2 | 3 | 4 | 5 | 6 | 7 |
| 5. I compare the past culture of the art with present-day culture.  **Porównuję sztukę dawną**  **ze współczesną.** | 1 | 2 | 3 | 4 | 5 | 6 | 7 |
| 6. I see the work of art as an extension of its time period.  **Postrzegam dzieło sztuki**  **jako przedłużenie epoki, z której pochodzi.** | 1 | 2 | 3 | 4 | 5 | 6 | 7 |
| 7. I try to place the work of art in its historical context.  **Staram się umiejscowić dzieło sztuki w kontekście historycznym.** | 1 | 2 | 3 | 4 | 5 | 6 | 7 |
| 8. I relate it to other works of art.  **Widzę powiązania między tym dziełem a innymi dziełami sztuki**. | 1 | 2 | 3 | 4 | 5 | 6 | 7 |
| 9. The composition of a work of art is important to me.  **Kompozycja dzieła sztuki**  **jest dla mnie ważna.** | 1 | 2 | 3 | 4 | 5 | 6 | 7 |
| 10. The colors of the work of art are important to me.  **Kolorystyka dzieła**  **jest dla mnie ważna.** | 1 | 2 | 3 | 4 | 5 | 6 | 7 |
| 11. I focus on the subtle aspects of the work of art.  **Skupiam się na subtelnych aspektach dzieła sztuki.** | 1 | 2 | 3 | 4 | 5 | 6 | 7 |
| 12. I try to understand the work completely.  **Staram się w pełni zrozumieć dzieło sztuki.** | 1 | 2 | 3 | 4 | 5 | 6 | 7 |
| 13. I try to understand what the artist is trying to communicate.  **Próbuję zrozumieć,**  **co artyst(k)a stara się przekazać.** | 1 | 2 | 3 | 4 | 5 | 6 | 7 |
| 14. I gain new insights about the work of art itself.  **Zdobywam nowe spojrzenie na to samo dzieło sztuki.** | 1 | 2 | 3 | 4 | 5 | 6 | 7 |
| 15. I see the work of art as an extension of the artist.  **Postrzegam dzieło jako przedłużenie artysty.** | 1 | 2 | 3 | 4 | 5 | 6 | 7 |
| 16. I have a clear idea of what to look for when viewing the work of art.  **Mam jasną wizję tego,**  **na co zwracać uwagę podczas oglądania dzieła sztuki**. | 1 | 2 | 3 | 4 | 5 | 6 | 7 |
| 17. I usually feel that my thoughts on the work of art are correct.  **Zwykle czuję, że moje przemyślenia na temat dzieła sztuki są słuszne.** | 1 | 2 | 3 | 4 | 5 | 6 | 7 |
| 18.I feel I am able to understand the work of art.  **Czuję, że jestem w stanie zrozumieć dzieło sztuki.** | 1 | 2 | 3 | 4 | 5 | 6 | 7 |
| 19. I lose track of time when I view the work of art.  **Tracę poczucie czasu,**  **kiedy oglądam dzieło sztuki.** | 1 | 2 | 3 | 4 | 5 | 6 | 7 |
| 20. I get lost in thought when I view the work of art.  **Zatracam się w myślach,**  **gdy odbieram dzieło sztuki.** | 1 | 2 | 3 | 4 | 5 | 6 | 7 |
| 21. I am completely focused on viewing the work of art.  **Jestem całkowicie skupiona/y na odbiorze dzieła sztuki.** | 1 | 2 | 3 | 4 | 5 | 6 | 7 |
| 22. The experience of viewing the work of art is rewarding to me.  **Doświadczenie oglądania dzieła sztuki jest dla mnie satysfakcjonujące.** | 1 | 2 | 3 | 4 | 5 | 6 | 7 |

ENG **Calculation of results**

Respondents respond to each statement on a 7-point Likert scale, from 1 (strongly disagree) to 7 (strongly agree). The results for each scale are obtained by adding together the points obtained by the tested person. The overall score is the sum of the points obtained by the subject in all scales. The questionnaire does not contain inverted questions. Factors:

- the **emotional** dimension includes statements 1, 2, 3, 4,
- the **cultural** dimension includes statements 5, 6, 7, 8,
- the **perceptual** dimension includes statements 9, 10, 11,
- the **understanding** dimension includes statements 12, 13, 14, 15,
- flow dimension (**proximal conditions**) contains theorems 16, 17, 18,
- flow dimension (**flow experience**) contains propositions 19, 20, 21, 22.

PL **Obliczanie wyników**

Badani ustosunkowują się do każdego twierdzenia na 7-punktowej skali Likerta, od 1 (zdecydowanie się nie zgadzam) do 7 (zdecydowanie się zgadzam). Wyniki dla każdej ze skal otrzymuje się poprzez dodanie do siebie punktów uzyskanych przez osobę badaną. Wynik ogólny jest sumą punktów uzyskanych przez badanego we wszystkich skalach. Kwestionariusz nie zawiera pytań odwróconych. Czynniki:

- wymiar **emocjonalny** zawiera stwierdzenia 1, 2, 3, 4,
- wymiar **kulturowy** zawiera stwierdzenia 5, 6, 7, 8,
- wymiar **percepcyjny** zawiera stwierdzenia 9, 10, 11,
- wymiar **zrozumienia** zawiera stwierdzenia 12, 13, 14, 15,
- wymiar **przepływu (warunki proksymalne)** zawiera twierdzenia 16, 17, 18,
- wymiar **przepływu (doświadczenie flow)** zawiera twierdzenia 19, 20, 21, 22.
